# Increasing Leaf Vein Density via Mutagenesis in Rice Results in an Enhanced Rate of Photosynthesis, Smaller Cell Sizes and Can Reduce Interveinal Mesophyll Cell Number

**DOI:** 10.3389/fpls.2017.01883

**Published:** 2017-11-01

**Authors:** Aryo B. Feldman, Hei Leung, Marietta Baraoidan, Abigail Elmido-Mabilangan, Irma Canicosa, William P. Quick, John Sheehy, Erik H. Murchie

**Affiliations:** ^1^Crops for the Future, Semenyih, Malaysia; ^2^Plant Breeding, Genetics and Biotechnology, The International Rice Research Institute, Los Baños, Philippines; ^3^The C_*4*_ Rice Center, The International Rice Research Institute, Los Baños, Philippines; ^4^Department of Animal Plant Sciences, University of Sheffield, Sheffield, United Kingdom; ^5^Division of Plant and Crop Sciences, School of Biosciences, University of Nottingham, Sutton Bonington, United Kingdom

**Keywords:** photosynthesis, leaf anatomy, venation, mutation breeding, rice

## Abstract

Improvements to leaf photosynthetic rates of crops can be achieved by targeted manipulation of individual component processes, such as the activity and properties of RuBisCO or photoprotection. This study shows that simple forward genetic screens of mutant populations can also be used to rapidly generate photosynthesis variants that are useful for breeding. Increasing leaf vein density (concentration of vascular tissue per unit leaf area) has important implications for plant hydraulic properties and assimilate transport. It was an important step to improving photosynthetic rates in the evolution of both C_3_ and C_4_ species and is a foundation or prerequisite trait for C_4_ engineering in crops like rice (*Oryza sativa*). A previous high throughput screen identified five mutant rice lines (cv. IR64) with increased vein densities and associated narrower leaf widths (Feldman et al., 2014). Here, these high vein density rice variants were analyzed for properties related to photosynthesis. Two lines were identified as having significantly reduced mesophyll to bundle sheath cell number ratios. All five lines had 20% higher light saturated photosynthetic capacity per unit leaf area, higher maximum carboxylation rates, dark respiration rates and electron transport capacities. This was associated with no significant differences in leaf thickness, stomatal conductance or CO_2_ compensation point between mutants and the wild-type. The enhanced photosynthetic rate in these lines may be a result of increased RuBisCO and electron transport component amount and/or activity and/or enhanced transport of photoassimilates. We conclude that high vein density (associated with altered mesophyll cell length and number) is a trait that may confer increased photosynthetic efficiency without increased transpiration.

## Introduction

Genetic gains in the grain yield per hectare of major crops like rice and wheat need to rise faster than the current rate in order to meet the needs of a growing global population and the negative effects of climate change (Ray et al., 2012; Challinor et al., 2014). Leaf photosynthesis has been identified as an important trait because it is an essential component of canopy radiation-use efficiency which currently operates well below the theoretical maximum (Murchie et al., 2009; Ort et al., 2015). It has been shown empirically and theoretically that an improvement in photosynthetic capacity and quantum yield will raise canopy photosynthesis, biomass and yield, especially under optimal conditions (Long et al., 2006; Mitchell and Sheehy, 2006; Murchie et al., 2009). Photosynthesis has many components that could be improved, such as Calvin Cycle activity and kinetics, and photoprotection (Murchie and Niyogi, 2011; Kromdijk et al., 2016). In the case of rice, high tropical temperatures have attracted research into the feasibility of introducing C_4_ mechanisms (Karki et al., 2013; Covshoff et al., 2016). However, substantial gains can also be made through altering the existing C_3_ pathway (Zhu et al., 2010; Ort et al., 2015).

Leaf venation is a trait that underlies several important physiological responses and provides mechanical support (Sack and Scoffoni, 2013). However, it may have been overlooked in terms of breeding. The reduced interveinal density (IVD), or high vein density, characteristic is thought to be one of the earliest steps in the evolution of C_4_ photosynthesis by many researchers (McKown and Dengler, 2007, 2009; Kajala et al., 2011; Christin et al., 2013). However, leaf venation also played crucial roles in terrestrial plant evolution long before the rise of C_4_ photosynthesis. It is key to the plant hydraulic system as water molecules move much less easily through mesophyll cells (MCs) (probably via the apoplastic pathway) compared to how they are conducted through the xylem (via tracheids and vessels) (McElrone et al., 2013). The venation system should be tightly linked to photosynthesis since CO_2_ enters the plant via the stomata in exchange for transpired water (around one molecule of the former for 400 molecules of the latter, McElrone et al., 2013) and because photosynthate transport away from the leaf is via the phloem. Therefore, good vascular connectivity is needed for MCs to support high photosynthesis (Brodribb et al., 2007). This would seem to justify the observation that evolutionary changes in vein density are positively correlated with higher maximum photosynthesis (A_max_) values. The three-to-four-fold increase in vein density during early angiosperm evolution has been connected to the rise in photosynthetic capacity (as well as transpiration and hydraulic efficiency) in the absence of other C_4_ traits (Boyce et al., 2009; Brodribb and Feild, 2010).

Leaf vein density in cereals is a readily measured trait that may provide potential for improving photosynthesis via its effects on hydraulic conductance. The relationship between plant hydraulic conductance and photosynthesis is clear as shown for rice (Taylaran et al., 2011). However, the correlation between leaf vein density and leaf hydraulic conductance is not consistently shown in the literature and recent work in the natural variation in the dicot, *Arabidopsis thaliana*, failed to show a relationship between photosynthesis and vein density (Nardini et al., 2003; Scoffoni et al., 2011; Rishmawi et al., 2017).

The following investigation into the phenotypes of novel rice mutant variants shows how increased leaf vein density confers beneficial photosynthetic traits that are not directly associated with the C_4_ condition but which may provide resources for improving C_3_ photosynthesis and for C_4_ engineering. A deeper understanding of the increased vein density trait itself and how it links to other leaf anatomy traits (Giuliani et al., 2013) to improve both C_3_ and C_4_ photosynthesis will further benefit efforts toward raising the photosynthetic capacity and efficiency of rice (and other candidate C_3_ crops) and so improving future agricultural production.

## Materials and Methods

### Plant Material

The primary genetic resource was IRRI IR64 mutant library (consisting of 66,891 lines) that was derived from a single rice plant (*Oryza sativa* cv. IR64), IR64-21 (Wu et al., 2005). The mutant library, as for all IRRI seed stock, follows all the international guidelines and legislation of the International Treaty on Plant Genetic Resources for Food and Agriculture. The original IR64-21 parent plant was selected for phenotypic uniformity from breeder seeds grown under field conditions. IR64 mutants used in the screening were either deletions (mainly dry seeds mutagenized with γ-radiation at 250 GY at the International Atomic Energy Agency) or point mutations (mainly pre-soaked seed mutagenized with EMS at 0.4, 0.6, 0.8, 1.0, and 1.6% concentrations at IRRI). Mutagenized lines were advanced every 4 months from the initial M_1_ (first mutant generation) population by single seed descent until at least the M_3_ generation. This was to ensure near homozygous inbred lines, which was essential for quantifying phenotypic characteristics in replicated trials.

Increased vein density lines were selected using a high throughput screen (Feldman et al., 2014). Rice plants from the deletion mutant populations had their veins counted on-site using a handheld microscope (Readiview handheld microscope, Meade Instruments Corporation, Irvine, CA, United States) at 80x magnification.

### Leaf Anatomy

High vein density lines from the mutant populations were subjected to detailed microscopy analysis. Vein counts per 2 mm and cellular arrangements were viewed with a light microscope (Olympus BX51 Motorized Research Microscope, Tokyo, Japan, connected to an Olympus DP71 Microscope Digital Camera, Tokyo, Japan). Images were taken using Olympus CellˆP imaging software (from the Olympus Cell^∗^ software family) and had their parameters quantified using ImageJ (Version 1.44).

**FIGURE 1:**
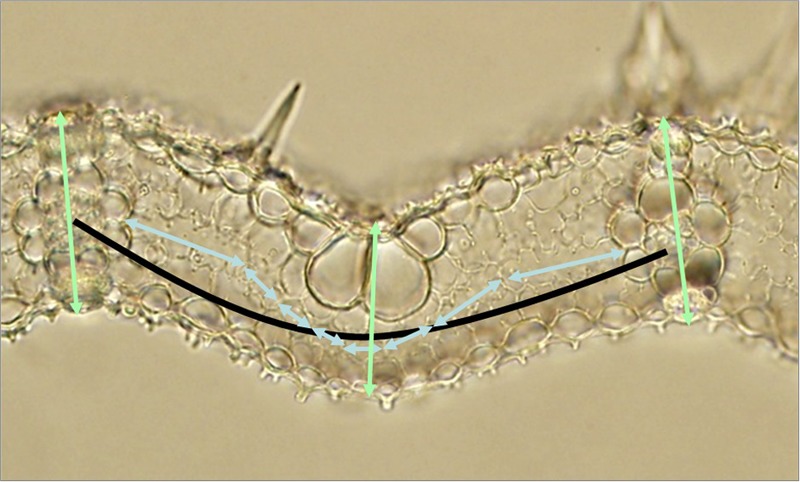
Determining anatomical parameters in a rice leaf section. The light blue double-ended arrows represent mesophyll cell lengths (and their number equates to the mesophyll number). The light green double-ended arrows represent leaf thicknesses. The black arc represents interveinal distance. Measurements of line length were made using a calibrated microscope and imageJ as described in “Materials and Methods”.

Vein density viewings were taken of fresh leaf samples at 4x magnification. Samples were then fixed in FAA (formalin-acetic acid-alcohol solution) (500 ml absolute ethanol : 270 ml formaldehyde : 50 ml acetic acid : 180 ml water) for at least 12 h. These were cross-sectioned by hand using a razor blade. Leaf sections were placed in 85% ethanol for 24 h, after which the ethanol was removed and rinsed off with water. A fresh application of 85% ethanol was then applied for 24 h. The ethanol was removed and rinsed off with water after this period and replaced by lactic acid for 30–36 h before being washed with water. At 40x magnification, MCs and BSCs could be viewed. Measurements of leaf thickness, IVDs, numbers of MCs between veins and MC lengths were made for leaf 5–7 of three replicate plants per line. **Figure 1** illustrates how these parameters could be taken from leaf section images.

### Photosynthesis

Photosynthesis measurements were performed using the portable IRGAs, LI-COR LI-6400 and LI-6400XT machines (LI-COR Environmental, Lincoln, NE, United States). A/C_i_ [assimilation rate (μmol CO_2_ m^-2^ s^-1^) over intercellular CO_2_ concentration (μmol mol^-1^)] curves were run according to the following program: CO_2_R (CO_2_ reference) steps (in order) of 1800, 1800, 1600, 1400, 1200, 1000, 800, 600, 400, 200, 100, 80, 60, 40, 20, and 1800 μmol mol^-1^ with 3–5 min waiting time, 1500 μmol m^-2^ s^-1^ PAR (photosynthetically active radiation), 400 μmol s^-1^ gas flow and 30°C block temperature. The program descended in CO_2_R levels for each subsequent step (besides the second and last ones) as it was found from trial testing that it took the leaf longer to adjust to increasing, as opposed to decreasing, ambient CO_2_ levels. The first step of 1800 μmol mol^-1^ [CO_2_R] was repeated to ensure that the leaf had enough time to stably react to the high [CO_2_]. The last step of 1800 μmol mol^-1^ [CO_2_R] was included to prepare the photosynthesis machine for the next round of measurements.

A/PAR [assimilation rate over incident PAR (μmol photons m^-2^ s^-1^)] curves were run according to the following program: PAR steps (in order) of 2000, 1750, 1500, 1250, 1000, 750, 500, 250, 100, 50, 20, 0, and 2000 μmol m^-2^ s^-1^ with 3–5 min waiting time, 400 μmol mol^-1^ CO_2_R, 400 μmol s^-1^ gas flow and 30°C block temperature. The program descended in PAR levels for each subsequent step (besides the last one) as it was found from trial testing, that it took the leaf longer to adjust to increasing, as opposed to decreasing, levels. The last step of 2000 μmol m^-2^ s^-1^ PAR was included to prepare the photosynthesis machine for the next round of measurements and was not used in data analysis.

Cuvette humidity levels were always kept between 67 and 70%. Plants were kept well-watered during the photosynthesis measurements to ensure that stomatal conductance was always high (stomatal conductance at ambient CO_2_ level was ≥0.4 mol m^-2^ s^-1^).

Three replicate measurements of the same leaf (the youngest fully expanded leaf of the main tiller) of a plant at mid-vegetative stage were taken using the same IRGA machine for each photosynthesis curve of each plant measured. Controlling for variation with time, rather than sibling plants per line, was found to be the most important factor and was controlled by measuring the same leaf/same plant/same line/mutant without adding variation with intra-genetic-line variation. Measurements on control wild-type plants were made in a way that effectively accounted for time, environment and developmental variation. Measurements took place between 08:30 and 14:30 under greenhouse conditions. Plants were treated with artificial lighting that was controlled to give a light intensity of 200 μmol m^-2^ s^-1^ when days were particularly overcast.

Photosynthetic parameters were calculated both manually and through modeling and curve fitting software (Sharkey et al., 2007). From an A/C_i_ curve, the CP (μmol mol^-1^) was the x-axis intercept of the regression line for photosynthesis rates when C_i_ was <200 μmol mol^-1^ (six data points). The model used was based on the Farquhar and von Caemmerer biochemical model of leaf photosynthesis and calculated the remaining parameters, i.e., Vc_max_ (rate of RubisCO-catalyzed carboxylation) (μmol m^-2^ s^-1^), J_max_ [the regeneration of RuBP (ribulose-1,5-bisphosphate) controlled by the rate of TPU) (μmol m^-2^ s^-1^], TPU (capacity of the leaf to use the primary product of the chloroplast) (μmol m^-2^ s^-1^), g_m_ (mesophyll conductance) (μmol m^-2^ s^-1^ Pa^-1^).

From an A/PAR curve, R_d_ (μmol CO_2_ mol^-1^) was the negative y-axis intercept of the regression line for photosynthesis rates when PAR was <200 μmol m^-2^ s^-1^ (four data points). The slope of the regression line was taken to be the maximum QE. The photosynthesis rate at saturating PAR was taken to be A_max_. Instantaneous WUE (assimilation rate divided by transpiration rate; A/E) and intrinsic WUE (assimilation rate divided by stomatal conductance rate; A/g_s_) were calculated at A_max_.

### Growing Conditions

Measurements took place at the IRRI greenhouses (14° 10′ 23.29″, 121° 15′ 32.44″). Seeds were incubated at 50°C for 72 h if their dormancy had to be broken, i.e., if seeds were more than 2 years old or if germination rates were particularly poor (<30%). They were then pre-germinated in petri dishes on filter paper treated with sterilized distilled water and incubated at 35°C until the radical and coleoptile was sufficiently swollen (usually by 24 h). Soil was sourced from the IRRI upland farm and was treated with 0.09–0.01–0.09 g NPK fertilizer per kg soil as a basal dressing and 0.09 g N fertilizer per kg soil every 2–3 weeks, depending on plant growth rate (determined qualitatively on a relative scale). Irrigation was applied generously to ensure that the soil moisture content was always high.

### Statistical Analysis

One way analyses of variance (ANOVAs) tested differences between categories (e.g., rice genotype) for dependent variables (e.g., vein density, leaf width and anatomical traits). Simple linear regressions tested whether two dependent variables were correlated. Regression analyses and ANOVAs were calculated using Genstat Twelfth Edition (Version 12.1.0.3278).

## Results

### Linkage of Vein Density with Leaf Width

In a previous study, it became apparent that variants that had narrower leaf widths were more likely to have higher vein densities (Feldman et al., 2014). This led to a candidate list of narrow leaf mutants, which both segregated for the increased vein density trait close to an expected ratio (1:3 for a monogenic recessive gene) and transmitted the phenotype over successive generations.

**FIGURE 2:**
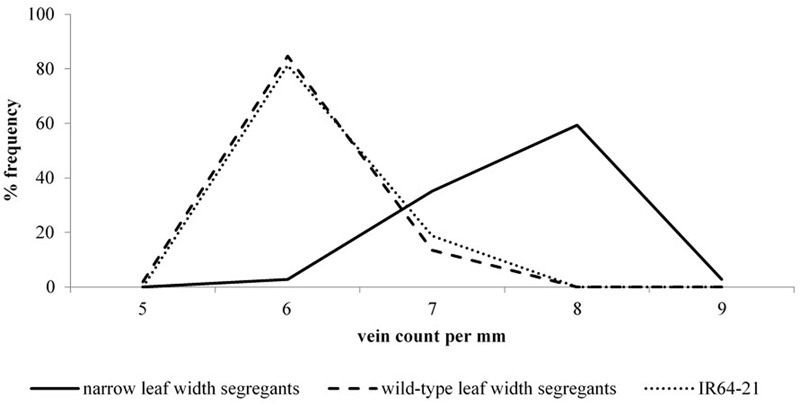
Vein density frequency curves for narrow and wild-type leaf width BC_1_F_2_ deletion mutant segregants. BC_1_F_2_: second filial generation after one backcross. Distribution frequencies illustrate the relative number of plants for each vein density in the IR64 wild-type (dashed line), wild-type leaf width mutant segregants (dotted line) and narrow leaf width segregants (solid line).

It was clear that leaf width and vein density were also linked in BC_1_F_2_ (second filial generation after one backcross) segregants of the high vein density, narrow leaf width mutant lines (**Figure 2**). Segregants with the wild-type leaf width phenotype predominantly had wild-type vein density phenotypes. The opposite was the case for the remaining segregants, which most often had narrow leaves and high vein densities. It can be seen in **Figure 2** that the wild-type leaf width segregants had almost the same frequency distribution of vein densities as for the IR64 wild-type. This was unlike the mutant leaf width segregants, which had a distinct frequency distribution curve for vein densities that peaked at 2 veins mm^-1^ greater than the peak of the controls (wild-type leaf width segregants and IR64 wild-type).

**FIGURE 3:**
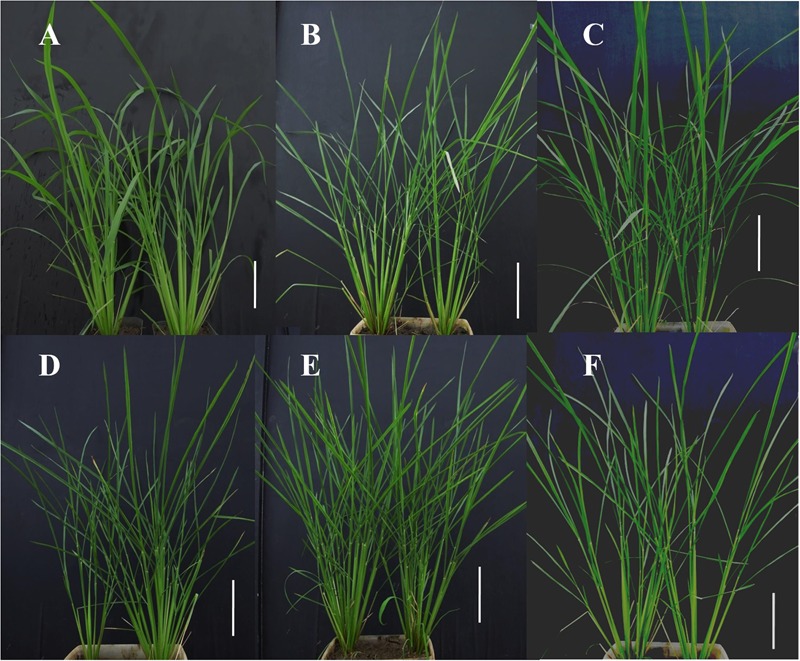
Phenotyping images of M_5_ mutant progeny at the mid-vegetative stage. M_5_: fifth mutant generation. White scale bar: 15 cm. **(A)** Wild-type IR64, #1 and #2. **(B)** E19076-1-5-3, #1 and #2. **(C)** E22097-1-3-1, #1 and #2. **(D)** E26181-1-1-2, #1 and #2. **(E)** Ell068-1-10-1, #1 and #2. **(F)** G558-11-5-2, #1 and #2.

**FIGURE 4:**
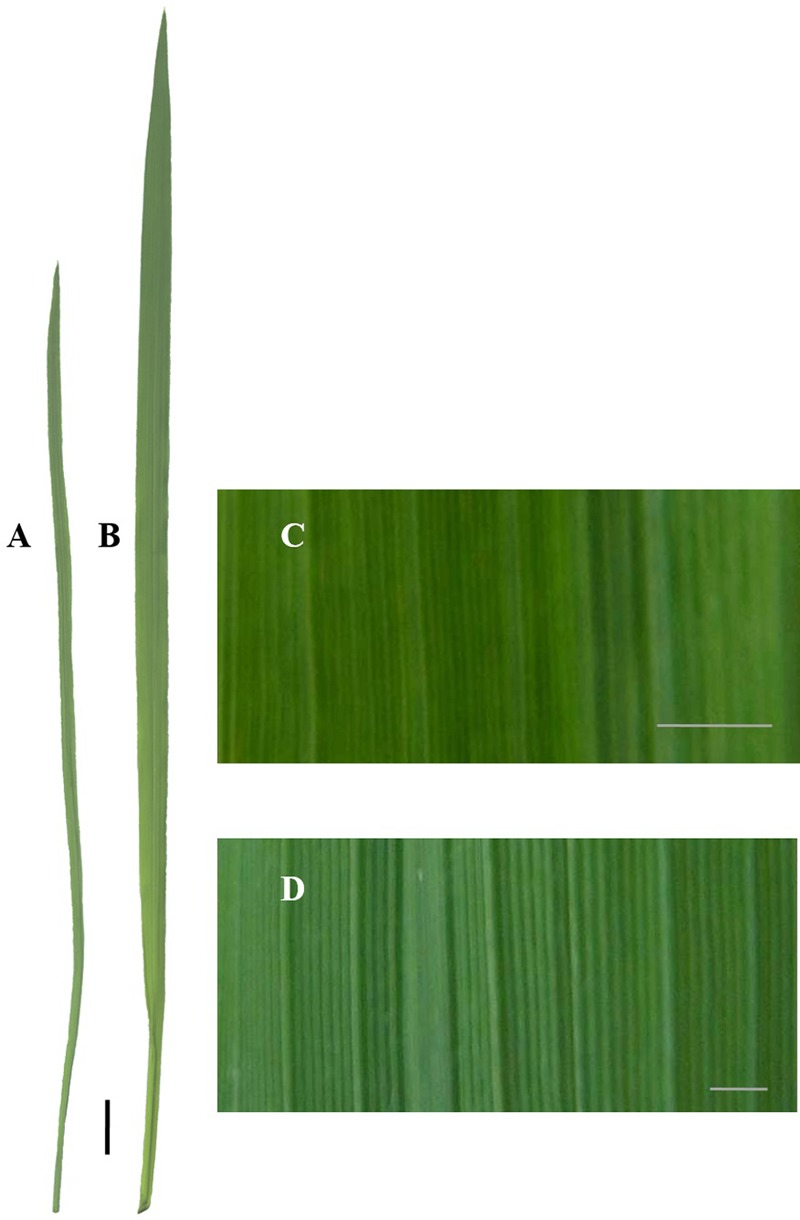
Comparing mutant and wild-type leaf phenotypes in BC_1_F_2_ line, E19076-1-5-1/IR64-21 at the mid-vegetative stage. BC_1_F_2_: second filial generation after one backcross. Black scale bar: 1 cm. White scale bars: 1 mm. **(A)** Mutant segregant leaf. **(B)** Wild-type segregant leaf. **(C)** Mutant segregant leaf surface. **(D)** Wild-type segregant leaf surface.

**Figure 3** illustrates the level of phenotypic distinction of the M_5_ candidate lines (**Figures 3B–F**) from the IR64 wild-type (**Figure 3A**). This phenotypic distinction was equivalent to that between mutant and wild-type segregants in the BC_1_F_2_ mutant candidate lines. Though plants were qualitatively not much shorter and had similar numbers of tillers, mutants had distinctly narrow leaf sheaths in addition to narrow leaf blades. This also made their stems narrower. The degree to which mutant plants had narrower leaves is illustrated in **Figure 4**. Line E22097-1-3-1 (**Figure 3C**) had the widest leaves of the mutants: 9 mm for the widest section of the widest leaf compared to 5–6 mm for other mutant lines and 11 mm for the parental control.

The qualitative scoring of the narrow leaf trait in mutant plants was straightforward and reliable. In addition to having narrow stems and leaves, they had a darker green color (stems and leaves) and had less leaf ridges (which could be sensed both tactilely and visually) (**Figure 4**).

### Identifying Changes in the Cellular Arrangement of Leaf Anatomy

The vein density screen was partly set up to test the hypothesis that increases in vein density would lead to changes in leaf cell structure, fundamentally in terms of decreasing interveinal MC numbers (for a low MC to BSC ratio), which is especially important to C_4_ photosynthesis as the two cell types perform separate functions in C_4_ photosynthesis.

Increased vein densities in C_4_ plants are the direct result of a leaf anatomy that has smaller IVDs and fewer MCs of approximately the same size, compared to C_3_ plants. The link between vein density and cellular arrangement in the *Oryza* genus was confirmed in various rice lines (high vein density deletion mutants, non-high vein density deletion mutants, and ssp. *indica* and ssp. *japonica* wild-type cultivars) in a long-term greenhouse experiment (Supplementary Table S1). Vein density correlated negatively with IVD (*p* < 0.001) and negatively with MC number (*p* < 0.001) but did not correlate with MC length (*p* > 0.05). MC number was positively correlated with IVD (*p* < 0.001) and was negatively correlated with MC length (*p* < 0.005). Finally, MC length correlated positively with IVD (*p* < 0.001). Similar trends were found in a subsequent experiment of high vein density mutants (**Table 1**), except that rather than vein density correlating with MC number (*p* > 0.05), it correlated negatively with MC length (*p* < 0.001).

**Table 1:** Anatomical characteristics of M_5_ deletion lines.

IR64 family	Vein no. (mm^-1^)	Interveinal MC no.	MC length (μm)	IVD (μm)
E11068-1-10-1	6.78 ± 0.06^∗∗∗^	6.85 ± 0.21	15.81 ± 0.42^∗∗∗^	144.40 ± 2.50^∗∗∗^
E19076-1-5-3	6.80 ± 0.06^∗∗∗^	5.85 ± 0.20	19.00 ± 0.42^∗∗∗^	152.13 ± 5.09^∗∗∗^
E22097-1-3-1	6.70 ± 0.11^∗∗∗^	6.12 ± 0.17	18.46 ± 0.33^∗∗∗^	142.14 ± 3.07^∗∗∗^
E26181-1-1-2	6.83 ± 0.11^∗∗∗^	5.77 ± 0.18^∗^	17.85 ± 0.35^∗∗∗^	134.00 ± 2.50^∗∗∗^
G558-11-5-2	6.49 ± 0.07^∗∗∗^	5.69 ± 0.15^∗^	18.67 ± 0.31^∗∗∗^	137.17 ± 3.11^∗∗∗^
All mutants	6.77 ± 0.07^∗∗∗^	6.01 ± 0.09	18.47 ± 0.16^∗∗∗^	141.04 ± 1.54^∗∗∗^
Wild-type	5.85 ± 0.04	6.30 ± 0.19	23.17 ± 0.46	182.00 ± 3.87

*Values are means ± SE of the fifth to seventh leaves of three replicate plants. ^∗^Significantly different from the IR64 wild-type at *p* < 0.05. ^∗∗∗^Significantly different from the IR64 wild-type at *p* < 0.001.*

**FIGURE 5:**
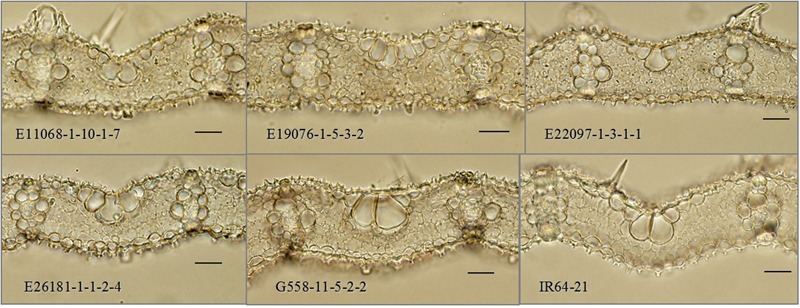
Cross-sections of interveinal leaf areas in M_5_ mutant lines. M_5_: fifth mutant generation. Black scale bars: 50 μm.

Mutant lines in the final candidate deletion mutant population (E11068-1-10, E19076-1-5, E22097-1-3, E26181-1-1 and G558-11-5) were not only distinguished by their higher vein densities and narrower leaf widths, but were also distinct in terms of cellular structure. They had smaller values of IVD and MC length compared to their parental control (*p* < 0.001) (**Figure 5** and **Table 1**). MC counts were generally not different from the control with the exceptions of E216181-1-1-2 and G558-11-5-2, both of which had fewer MCs (*p* < 0.05).

### Photosynthesis

**FIGURE 6:**
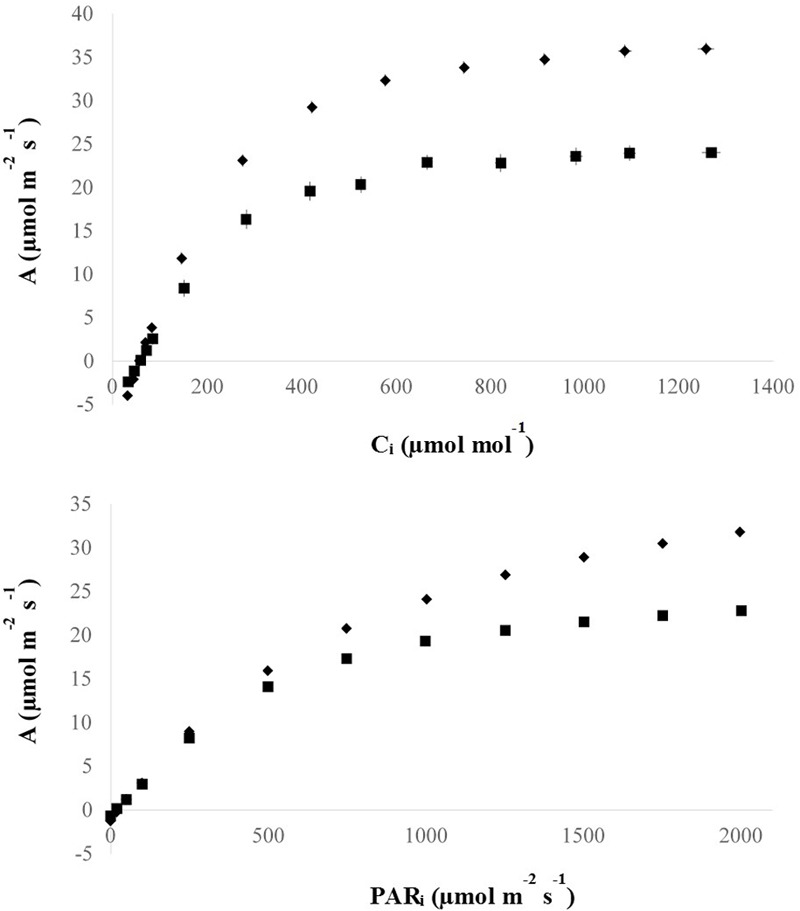
Photosynthesis curves of M_5_ mutant lines. M_5_: fifth mutant generation. Mutant data points represent means (diamonds), and horizontal and vertical lines represent standard *x*-axis (means of 10.30 μmol mol^-1^ and 0.57 μmol m^-2^ s^-1^ for A/C_i_ and A/PAR curves, respectively) and *y*-axis (means of 0.97 and 0.13 μmol m^-2^ s^-1^ for A/C_i_ and A/PAR curves, respectively) error bars, respectively, of three replicate measurements of the same leaf of the same plant for five lines (*n* = 15). Wild-type data points represent means (squares), and horizontal and vertical lines represent standard *x*-axis (means of 12.09 μmol mol^-1^ and 0.56 μmol m^-2^ s^-1^ for A/C_i_ and A/PAR curves, respectively) and y-axis (means of 1.25 and 0.20 μmol m^-2^ s^-1^ for A/C_i_ and A/PAR curves, respectively) error bars, respectively, of five replicate measurements of the same leaf of one replicate plant and three replicate measurements of the same leaf of another replicate plant (*n* = 8). It should be noted that there are error bars that are so small that they are hidden by mean point symbols.

Apart from the association with other C_4_ metabolic adaptations, vein density has also been linked to photosynthetic performance possibly by controlling the capacity of the hydraulic system and photosynthate transport (Brodribb et al., 2007). To help test this, photosynthetic rates of high vein density candidate mutant plants were measured at varying CO_2_ and light levels (**Figure 6**). In this way, candidate mutants could be characterized by key photosynthetic parameters that distinguish C_4_ from C_3_ species (**Table 2**). No C_4_–specific properties were found in these plants.

**Table 2:** Photosynthetic characterization of M_5_ deletion candidate lines.

	CO_2_ CP	Vc_max_^∗^	J_max_^∗∗∗^	TPU^∗∗∗^	g_m_	R_d_^∗∗∗^
	(μmol mol^-1^)	(μmol m^-2^ s^-1^)	(μmol m^-2^ s^-1^)	(μmol m^-2^ s^-1^)	(μmol m^-2^ s^-1^ Pa^-1^)	(μmol mol^-1^)
All mutants	58.23 ± 1.97	181.41 ± 10.83	174.28 ± 6.06	13.15 ± 0.43	16.83 ± 3.00	1.12 ± 0.08
Wild-type	54.04 ± 2.99	127.68 ± 9.90	115.91 ± 8.41	8.54 ± 0.61	19.67 ± 4.88	0.61 ± 0.08

	**QE**	**A_max_^∗∗∗^**	@**C_i_**	@**g_s_^∗∗^**	**A/g_s_**	**A/E**
	**(mol mol^-1^)**	**(μmol m^-2^ s^-1^)**	**(μmol mol^-1^)**	**(mol m^-2^ s^-1^)**	(**μmol mol^-1^)**	**(mmol mol^-1^)**
All mutants	0.04 ± 0.00	31.8 ± 1.02	293.89 ± 3.27	0.74 ± 0.03	43.55 ± 1.56	2.79 ± 0.09
Wild-type	0.04 ± 0.00	22.78 ± 1.07	292.86 ± 4.39	0.56 ± 0.04	41.91 ± 2.86	3.05 ± 0.10

*CO_*2*_ CP, carbon dioxide compensation point. Vc_*max*_, maximal carboxylation rate. J_*max*_, electron transport capacity. TPU, triose phosphate utilization rate. g_*m*_, mesophyll conductance. R_*d*_, dark respiration. QE, quantum efficiency. A_*max*_, maximum (light saturated) CO_*2*_ assimilation rate. C_*i*_, internal CO_*2*_ concentration. g_*s*_, stomatal conductance. E, evapotranspiration rate. Values for mutants are means ± SE of three replicate measurements of the same leaf of the same plant for five lines (*n* = 15). Values for wild-type are means ± SE of five replicate measurements of the same leaf of one IR64 parental plant and three replicate measurements of the same leaf of another replicate plant (*n* = 8). g_*m*_ values were constrained to be ≤30 (μmol m^-*2*^ s^-*1*^ Pa^-*1*^). ^∗^Significantly different from the wild-type at *p* < 0.05. ^∗∗^Significantly different from the wild-type at *p* < 0.005. ^∗∗∗^Significantly different from the wild-type at *p* < 0.001. Corresponding g_*s*_ and C_*i*_ values are given for each A_*max*_ figure as indicated by ‘@’. For a breakdown of photosynthetic parameters for ‘all mutants’ into individual lines, see Supplementary Table S2.*

**Figure 6** gives an overview of the A/C_i_ and A/PAR responses and shows that the mutant plants had higher (*p* < 0.001) photosynthesis for higher levels of incident light (PAR) and C_i_ compared to the IR64 wild-type control.

**Table 2** shows the results of photosynthetic analysis. From the A/C_i_ curve, Vc_max_ indicates the amount of active RuBisCO present in the leaf, J_max_ represents the capacity for thylakoid electron transport and TPU is an estimation of the ability to use the end-products of photosynthesis in the chloroplast. From the light response curve, A_max_ is the light saturated photosynthetic capacity and R_d_ is the rate of CO_2_ loss with or without light energy (i.e., respiration rate). All five parameters were increased in mutant lines (*p* < 0.05 for Vc_max_; *p* < 0.001 for J_max_, TPU, R_d_ and A_max_). Supplementary Table S2 illustrates variation between mutant lines for these parameters.

A plant’s leaf thickness can be correlated with photosynthetic capacity due to the accumulation of photosynthetic components per unit leaf area in C_3_ plants (Peng, 2000; Marenco et al., 2009). Additionally, thinner leaves are characteristic of C_4_ species where they ensure closer contact between MCs and BSCs (Dengler et al., 1994; Leegood, 2000) as well as a shorter vertical distance between veins and the stomatal epidermis. The leaf thickness of candidate lines was generally lower (*p* < 0.001, combining all lines) than the control, even though leaf thickness varied between mutant lines (*p* < 0.001). The leaf thickness of IR64-21 had an average of 66.46 ± 0.47 μm compared to 57.43 ± 0.66 μm for E26181-1-2, 59.71 ± 0.69 μm for E11068-1-10-1, 61.05 ± 0.78 μm for E19076-1-5-3, 62.93 ± 0.66 μm for G558-11-5-2 and 66.98 ± 1.07 μm for E22097-1-3-1.

## Discussion

Both the high vein density and narrow leaf width phenotypes were explained by reductions in leaf cell size without changes in interveinal cell number, in most mutant lines. However, MCs were both shorter in length and fewer in number in two of the five high vein density lines (E216181-1-1-2 and G558-11-5-2). Results showed a decrease in IVD, from 182 to as low as 134 μm, and in interveinal MC number, from 6.30 to as few as 5.69.

The relationship between leaf shape and anatomy deserves further attention to establish whether there is a common regulator of leaf width and vein density (Nelson and Dengler, 1997), such as the underlying anatomy or biochemical pathways [e.g., auxin (Dengler and Kang, 2001) and ethylene (Feng et al., 2015) regulation]. Leaf morphogenesis is not simply the sum of cell size and proliferation but is also affected by their interaction (Tsukaya, 2005), which is coordinated by at least three distinct mechanisms (Hisanaga et al., 2015). Various rice genotypes were shown to have a negative correlation between MC number and MC length (*p* < 0.005) (**Table 1** and Supplementary Table S1). This negative correlation was also evident in Tsukaya (2002) and Tsukaya (2003). The interaction could not have been a compensation mechanism as fewer cells can trigger increases in cell size but the inverse does not exist, and so rather a form of cell–cell communication is responsible for the interaction (Tsukaya, 2005). It should also be noted that the functional transition from proliferation (fixing cell number) to expansion (fixing cell size) is unidirectional and regulated by a host of genes (Kalve et al., 2014). Therefore, it may be possible that variants for decreased interveinal MC number could be modified to increase their MC sizes. It should be noted, however, that identifying rice mutants that have fewer but larger cells may be especially difficult. This is because the relatively small MC sizes of rice may be highly conserved for the reduction and recovery from photorespiration by maximizing chloroplast containing MC border surface area (Sage and Sage, 2009).

Even though the narrow leaf width phenotype was associated with the more important trait of high vein density (more specifically, a low MC:BSC ratio), it may not need to be removed from candidate lines by breeding. Having a narrow leaf width certainly was not detrimental to photosynthetic function as mutants generally outperformed their parental wild-type (**Table 2**, *p* < 0.001).

Photosynthesis per unit leaf area in plants is limited by a combination of prevailing factors and intrinsic leaf properties. For a leaf in optimal conditions, i.e., open stomata and saturating light, it is common for the rate of photosynthesis to be dominated by the activity of the enzyme, RuBisCO (with co-limitations by the rate of RuBP regeneration and stomatal conductance). The conductance of the leaf to CO_2_ and the leaf temperature are important in determining the ratio of activity of carboxylation and oxygenation within the chloroplast. Therefore, under the experimental measurement conditions, we might have expected RuBisCO activity to dominate. Our data show clearly that photosynthetic capacity per unit leaf area is higher in the mutant lines compared to the parental wild-type (**Table 2**, *p* < 0.001). The increase is associated with heightened end-product capacity (TPU), RuBisCO activity (higher Vc_max_) and electron transport capacity (J_max_). Vc_max_ values are important since they are associated with an increase in the amount of active RuBisCO protein, an increase in the activation state of existing protein and/or an increase in the relative carboxylation activity of the existing protein per unit leaf area. Our data did not permit us to identify which mechanism dominates here, although the increased TPU and Vc_max_ suggested that a combined increased capacity for both sucrose export and hydraulic conductance could be responsible (Brodribb et al., 2007; Brodribb and Feild, 2010). This would imply that the higher Vc_max_ arose from an increased activation state of existing RuBisCO protein. This is associated with a slightly higher stomatal conductance and lower leaf instantaneous water use efficiency (A/E), providing evidence for enhanced hydraulic capacity. g_m_ showed lower values in the mutant lines indicating that there was not a higher conductance for CO_2_.

Given that these measurements were made under saturating light, it will be important to understand how/whether this new morphology supports higher photosynthesis under conditions of moderate illumination, drought and varied atmospheric humidity. Although enhanced transpiration and lowered WUE can be considered a disadvantage in conditions of lowered water availability, the need for new traits in irrigated rice that enhance biomass productivity is of higher concern.

Takai et al. (2013) linked (genetically and physiologically) photosynthetic improvement in rice to changes in both leaf morphology (leaf width, thickness and shade of green) and anatomy (mesophyll cell density). The thinner leaves in our mutant lines may be a clue to explaining their improved photosynthetic performance. This is despite thicker leaves having been correlated with traits linked to higher photosynthetic capacity, such as chlorophyll content and photosynthetic enzyme activity (Peng, 2000; Marenco et al., 2009; Takai et al., 2013), and N concentration (Murchie and Horton, 2007; Zhao et al., 2008). Two mutant lines (E11068-1-10 and E19076-1-5) had decreased SLA values in the field (*p* < 0.001; results not shown and other mutant lines not measured) even though they had decreased leaf thicknesses when measured by microscopy. This was a surprising result given that the two parameters are generally closely negatively correlated (Wilson and Hattersley, 1989; Meziane and Shipley, 2001; Vile et al., 2005). The most likely explanation for the apparent inconsistency is that they had relatively high leaf weight densities, which would account for their low SLA values without the need for having thick leaves. Indeed, SLA varied with leaf density in various grasses rather than leaf thickness in Garnier and Laurent (1994), contrary to Li et al. (2005), and in fact higher leaf densities were shown to correlate with anatomical traits like a greater abundance of vascular tissue. If connecting this to the photosynthesis data, the thinner but denser leaves of mutant lines could be explained by a greater RuBisCO concentration, which would account for the increased Vc_max_ values. In addition, the observation of darker green leaves of candidate lines (**Figure 4C**) may have indicated a greater chlorophyll content, which should be confirmed in further studies.

## Conclusion

A previous high throughput screen identified five rice variants as having an increase in vein density, a foundational C_4_ photosynthesis trait (Feldman et al., 2014). These lines were shown to have other relevant traits: fewer interveinal MCs and enhanced photosynthetic rates. Therefore, given its low technology and cost, as well as its high throughput, the vein density screen could be applied to other plant species for identifying traits for the improvement of C_3_ photosynthesis as well as those relevant for the efforts to introduce CO_2_ concentrating mechanisms into rice.

## Availability of Data and Materials

Data supporting the article’s findings, which are not in the article itself, can be requested from the corresponding author.

## Author Contributions

The article’s authors, AF, HL, MB, AE-M, IC, WQ, JS, and EM made substantial contributions to the conception or design of the work (AF, HL, WQ, JS, and EM); or the acquisition (AF, MB, AE-M, and IC), analysis (AF, HL, and EM), or interpretation of data for the work (AF, HL, WQ, JS, and EM); and drafting the work or revising it critically for important intellectual content; and final approval of the version to be published; and agreement to be accountable for all aspects of the work in ensuring that questions related to the accuracy or integrity of any part of the work are appropriately investigated and resolved.

## Conflict of Interest Statement

The authors declare that the research was conducted in the absence of any commercial or financial relationships that could be construed as a potential conflict of interest. The reviewer RSC declared a shared affiliation with one of the authors, WQ, to the handling Editor.

## Abbreviations

A: CO_2_ assimilation rate
BC_1_F_2_: second filial generation after one backcross
BSC: bundle sheath cell
C_i_: intercellular CO_2_ concentrations
CO_2_R: CO_2_ reference
CP: compensation point
E: evapotranspiration rate
FAA: formalin-acetic acid-alcohol solution
g_m_: mesophyll conductance
g_s_: stomatal conductance rate
IRGA: infrared gas analyzer
IRRI: the International Rice Research Institute
IVD: interveinal distance
J_max_: the capacity for thylakoid electron transport
M_1_: first mutant generation
MC: mesophyll cell
PAR: photosynthetically active radiation
QE: quantum efficiency
R_d_: dark respiration
RuBisCO: ribulose bis-phosphate carboxylase/oxygenase
RuBP: ribulose-1,5-bisphosphate
SLA: specific leaf area
TPU: triose phosphate utilization
Vc_max_: rate of RubisCO-catalyzed carboxylation
WUE: water use-efficiency
