# Supplementary material for: Increasing Leaf Vein Density via Mutagenesis in Rice Results in an Enhanced Rate of Photosynthesis, Smaller Cell Sizes and Can Reduce Interveinal Mesophyll Cell Number
Source: Front Plant Sci. 2017 Nov 1;8:1883. doi: 10.3389/fpls.2017.01883 (PMC5672787; doi:10.3389/fpls.2017.01883)
Supplement: Supplementary file 1 [file Table_1.DOCX]

Supplementary Table S1. Anatomical characteristics of various rice lines***.*** Values are means ± SE of the fifth to seventh leaves of three replicate plants. *** Significantly different from the IR64 wild-type at p<0.001.

| **rice line categories** | **vein no. mm^-1^** | **interveinal MC no.** | **MC length (µm)** | **IVD (µm)** |
| --- | --- | --- | --- | --- |
| high vein density mutants | 12.88 ± 0.26*** | 6.51 ± 1.16*** | 12.38 ± 2.38*** | 159.78 ± 13.24*** |
| non-high vein density mutants | 10.47 ± 0.09 | 9.59 ± 0.17 | 15.61 ± 0.27 | 196.59 ± 3.5 |
| *japonica* wild-type cultivars | 10.08 ± 0.16 | 9.4 ± 0.66 | 15.85 ± 0.81 | 201.53 ± 8.61 |
| wild-type (*indica*) | 10.29 ± 0.21 | 10.1 ± 0.73 | 15.42 ± 0.84 | 204.61 ± 5.51 |
