# Supplementary material for: Increasing Leaf Vein Density via Mutagenesis in Rice Results in an Enhanced Rate of Photosynthesis, Smaller Cell Sizes and Can Reduce Interveinal Mesophyll Cell Number
Source: Front Plant Sci. 2017 Nov 1;8:1883. doi: 10.3389/fpls.2017.01883 (PMC5672787; doi:10.3389/fpls.2017.01883)
Supplement: Supplementary file 2 [file Table_2.DOCX]

Supplementary Table S2. Photosynthetic characteristics of vein density mutants***.*** Values are means SE of three replicate measurements of the same leaf of the same plant. * Significantly different from the wild-type the IR64-21 wild-type at p<0.05. ** Significantly different from the IR64-21 wild-type at p<0.005. *** Significantly different from the IR64-21 wild-type at p<0.001. Corresponding gs and Ci values are given for each Amax value.

| **IR64 line** | **CO_2_ CP** | **Vcmax** | **Jmax** | **A/g_s_** | **A/E** |
| --- | --- | --- | --- | --- | --- |
|  | **(μmol mol^-1^)** | **(mmol mol^-1^)** | **(μmol mol^-1^)** | **(μmol mol^-1^)** | **(mmol mol^-1^)** |
| E11068-1-10-1 | 57.74 ± 0.18 | 0.17 ± 0*** | 39.06 ± 0.66*** | 62.61 ± 1.59* | 3.81 ± 0.29 |
| E19076-1-5-1 | 51.18 ± 1.52 | 0.12 ± 0.00 | 35.58 ± 2.06* | 58.55 ± 1.18 | 3.45 ± 0.16 |
| E22097-1-3-1 | 50.01 ± 0.13 | 0.17 ± 0.00*** | 42.78 ± 2.72*** | 50.53 ± 3.07 | 3.43 ± 0.11 |
| E26181-1-1-2 | 64.30 ± 2.77 | 0.14 ± 0.01* | 37.56 ± 2.12** | 48.20 ± 5.18 | 2.80 ± 0.31 |
| G558-11-5-2 | 67.91 ± 1.31* | 0.10 ± 0.00** | 28.58 ± 1.91 | 40.59 ± 1.71 | 2.63 ± 0.12* |
| wild-type | 54.04 ± 2.99 | 0.11 ± 0.00 | 27.73 ± 0.78 | 50.42 ± 4.78 | 3.34 ± 0.19 |
|  | **Rd** | **QE** | **Amax** | **@Ci** | **@g_s_** |
|  | **(μmol mol^-1^)** | **(mol mol^-1^)** | **(μmol m^-2^ s^-1^)** | **(μmol mol^-1^)** | **(mol m^-2^s^-1^)** |
| E11068-1-10-1 | 0.59 ± 0.12 | 0.04 ± 0.00 | 32.56 ± 0.33*** | 292.57 ± 2.97 | 0.73 ± 0.03* |
| E19076-1-5-1 | 1.24 ± 0.15** | 0.04 ± 0.01 | 34.39 ± 1.12*** | 281.13 ± 2.27 | 0.67 ± 0.04 |
| E22097-1-3-1 | 0.96 ± 0.03 | 0.05 ± 0.00* | 35.96 ± 0.19*** | 283.79 ± 5.00 | 0.85 ± 0.07* |
| E26181-1-1-2 | 0.99 ± 0.13 | 0.04 ± 0.00 | 29.84 ± 2.23* | 300.58 ± 4.17 | 0.71 ± 0.10 |
| G558-11-5-2 | 1.01 ± 0.20* | 0.04 ± 0.01 | 26.24 ± 0.49* | 311.39 ± 3.02* | 0.73 ± 0.03* |
| wild-type | 1.41 ± 0.14 | 0.03 ± 0.00 | 24.48 ± 0.25 | 289.65 ± 5.80 | 0.58 ± 0.05 |
